# Management of Tuberculosis at El-Obeid Teaching Hospital During the Sudanese Armed Conflict: A Single-Center Descriptive Study

**DOI:** 10.7759/cureus.110882

**Published:** 2026-06-15

**Authors:** Amal Khalil Y Mohammed, Eldisugi Humida, Namarig Alhadi Hamid, Hussain G Ahmed

**Affiliations:** 1 Department of Medicine, Faculty of Medicine, University of Kordofan, El-Obeid, SDN; 2 Department of Medicine, El-Obeid International Hospital, El-Obeid, SDN; 3 Department of Medicine, El-Obeid Teaching Hospital, El-Obeid, SDN; 4 Department of Pathology, Prof Medical Research Consultancy Center, El-Obeid, SDN; 5 Department of Histopathology and Cytology, Faculty of Medical Laboratory Sciences, University of Khartoum, Khartoum, SDN

**Keywords:** infection, sudan, tb management, treatment protocol, tuberculosis

## Abstract

Background: Tuberculosis (TB) is a major global health issue, particularly in countries with armed conflict, a poor health-care system, and low-income populations. The purpose of this study was to describe the characteristics, diagnostic approaches, and reported barriers to healthcare access among TB patients managed at El-Obeid Teaching Hospital during three years of the armed conflict (2023 to 2026) in the war zone.

Methodology: Between April 2023 and April 2026, a descriptive, prospective hospital-based study was conducted in El-Obeid, the capital of North Kordofan State, Sudan. The study included all patients diagnosed with TB in El-Obeid Teaching Hospital during the period from April 15, 2023, to April 15, 2026.

Results: This study investigated 2193 TB patients, with 70% male patients and 30% female patients. The population was split 52% urban and 48% rural. Approximately 63% of patients began anti-TB medication later, within two to six months. Delays in treatment were caused by security concerns (42%), financial constraints (32%), a lack of healthcare resources (22%), and missed diagnoses (3%). The majority of patients reported a "new episode," followed by re-registered and multi-drug-resistant TB (MDR-TB) cases (90.6%, 8.8%, and 0.6%, respectively). Of the 13 MDR cases, 46% were new episodes, 38% were MDR, and 16% were reregistered. In 194 reregistered cases, 56% were recurring, while 44% were defaulters.

Conclusion: The incidence rates of TB are increasing with the extent of war duration, particularly among men. Coinfection with HIV was significantly rising among women.

## Introduction

According to the World Health Organization's (WHO) Global TB Report 2024, around 10.6 million people got tuberculosis (TB) in 2023, with 1.6 million deaths [1]. TB is the leading cause of mortality, particularly in high-risk populations such as those with human immunodeficiency virus (HIV) and diabetes [2].

TB remains a significant global health challenge, particularly in countries with armed conflict, where communities face socioeconomic determinants. These include poverty, malnutrition, overcrowding, limited access to quality healthcare, security barriers, financial barriers, and a deteriorated health system. The burden of TB in the European Region contributes to a large proportion of multidrug- and rifampicin-resistant TB (MDR/RR-TB), and malnutrition may aggravate TB outcomes [3]. Mycobacterium bacilli, which are responsible for TB, can be detected using a variety of methods, including smear microscopy, GeneXpert, and culture. A chest X-ray is an effective first-line screening technique for TB. However, it cannot be deemed definitively diagnostic of TB because it cannot distinguish it from other lung infections, lung cancer, and old healed TB lesions, and its findings can be obscured by other lung disorders [4]. The test is considered in the Sudan protocol for the diagnosis of TB. Chest X-rays are also used to diagnose pleural and pericardial effusions when smear microscopy results are negative or when TB is suspected in children, as well as in special situations such as miliary TB. CT scans can be effective for diagnosing Pott's disease and abdominal TB [5,6]. CT scans and MRIs are not generally advised since they are too expensive in countries such as Sudan during times of conflict [7]. Therefore, the present study aimed to assess the management of TB in a war zone during the Sudanese armed conflict, 2023-2026.

## Materials and methods

This was a prospective descriptive hospital-based study conducted at El-Obeid Teaching Hospital in North Kordofan State, Sudan, from April 2023 to April 2026.

Hospital enrollment process for TB patients

Patients were identified through daily screening of TB clinic registers, inpatient admission diagnoses, and laboratory results at El-Obeid Teaching Hospital, Sudan. Eligible patients were approached within 24-72 hours of confirmed TB diagnosis or treatment initiation. After the confirmation of the TB diagnosis, written informed consent was obtained. Following consent, each participant was assigned a unique study ID, and baseline data were collected using a standardized enrollment form. Follow-up assessments were conducted alongside routine TB clinic visits, in accordance with the study hospital’s schedule.

Patient enrollment begins immediately after a confirmed TB diagnosis. Potential participants were identified through routine hospital systems, including the TB clinic register (new episode), laboratory tests, previous records, and referral notes.

Sample size

The sample size comprises all TB patients across the three years of the Sudan War (2023-2026).

Inclusion criteria

All patients diagnosed with active TB (pulmonary and extrapulmonary, according to the study case definition) within the recruitment period, those receiving TB care at the study hospital, and those able to provide informed consent (or availability of a legally authorized representative for consent and assent where applicable) were included.

Exclusion criteria

Patients previously enrolled in the study (to avoid duplication), those who did not provide consent, attainable according to ethical requirements, and those not expected to remain reachable for follow-up (only if this is pre-specified in the protocol and ethically justified) were excluded.

Baseline data collection

Unique study participant IDs, demographic characteristics of the patients, clinical presentation, symptom duration, TB site (pulmonary/extra-pulmonary), diagnostic tests performed, and results (smear/GeneXpert/culture/drug susceptibility testing (DST) where available), a history of previous TB treatment, and coinfection with HIV were collected.

Follow-up

Enrolled patients were followed prospectively from diagnosis through the key milestones of standard TB management, with planned assessments that matched the study protocol. Follow-up data were gathered concurrently with routine TB care visits.

According to the WHO-revised terminology [8], a person with TB who is classified as a new case is referred to as a "new episode". A recurrent case is a person with TB who has previously been treated for TB, was pronounced cured or had their most recent course of TB therapy completed and has now been diagnosed with a new episode of TB. Relapse occurs when the original surviving bacteria reactivate because the first infection was not eradicated. "Re-registered" (for treatment) refers to a previously notified TB case who began treatment and took TB medications for at least one month but was not pronounced cured or completed. They have now begun a new course of therapy. Adherence refers to how closely a person's conduct (for example, taking medication) aligns to agreed-upon instructions from a healthcare provider. MDR-TB is caused by strains of M. tuberculosis that do not respond to rifampicin and isoniazid. According to the WHO global indicators description, the end-of-treatment outcome set contains the following: cured, completed, died, failed, defaulted (lost the follow-up), and transferred out with an uncertain outcome.

Treatment algorithm

Intensive Phase (Months 1-2)

Four first-line medications are administered daily: isoniazid, rifampicin, pyrazinamide, and ethambutol.

Continuation Phase (Months 3-6)

Two medications are administered daily: isoniazid and rifampicin.

Protocol adherence criteria

Direct Observation

Drugs are swallowed while being monitored by a skilled health care provider, community volunteer, or selected family member.

Missing Doses

Adherence is process-oriented; every missing dosage necessitates rapid follow-up via asp phone call or home visit.

Treatment Interruption

If a patient fails to collect TB treatment for more than two months in a row, they are considered a defaulter.

Follow-up milestones

Sputum smear microscopy was performed at the end of the second month (end of the intense phase), the fifth month, and the sixth month (end of treatment) to monitor conversion. Monthly pill counts are conducted to confirm process-oriented adherence and modify dosages.

The key endpoint of the DOTS program is the proportion of new and retreatment TB cases completing treatment, which is defined as either bacteriologically verified cured or clinically regarded therapy completed.

Ethical consent

Besides the fact that all patients presented seeking treatment in the only center bearing TB services, each participant was asked to sign a written consent form consenting to the use of their data in future research.

Statistical analysis

The collected data were first organized in a datasheet before being placed into IBM SPSS Statistics for Windows, Version 29 (Released 2022; IBM Corp., Armonk, New York, United States). Percentages, frequencies, and cross-tabulations and chi-square test results were computed. Odds ratios (ORs) considering 95% confidence intervals (95% CIs) were calculated to assess the impact of various risk factors on management outcomes. A p-value of < 0.05 was considered statistically significant.

## Results

This study examined 2,193 TB patients, whose ages ranged from 1 to 105 years, with a mean age of 37. Among the participants, 1,539 (70%) were male, and 654 (30%) were female. Most patients were aged 26-35 years, followed by 19-25, 36-45, >55, <18, and 46-55 years, representing 542/2193 (24.7%), 409 (18.7%), 371 (16.9%), 369 (16.8%), 267 (12.2%), and 235 (10.7%), respectively.

Approximately 1151 (52%) were urban residents and 1042 (48%) rural. Most female patients were urban residents (60%), whereas most male patients were rural (50.6%). There was a rise of TB among urban women (OR (95%CI) = 1.52 (1.2625 to 1.8299), p < 0.0001, z statistics =4.422).

Most patients attended within the year 2025 (41%), followed by 2024, 2023, and four months in 2026, representing 27%, 18%, and 14%, respectively. For the new cases of TB during the Sudan war, the year 2023 was used as a reference (2024 vs. 2023: OR = 1.55 (95% CI 1.12 to 2.14); 2025 vs 2023: OR = 1.89 (95% CI 1.39 to 2.58); 2026 vs 2023: OR = 1.44 (95% CI 0.97 to 2.14)). The overall increase in the incidence of TB during war ("increase" is reflected mainly in 2024 and 2025) is statistically significant, with a p-value < 0.001.

Of the 2193 patients, 1463/2193(%) were pulmonary TB patients, and the remaining 730 (%) were extrapulmonary TB patients.

**Figure 1:**
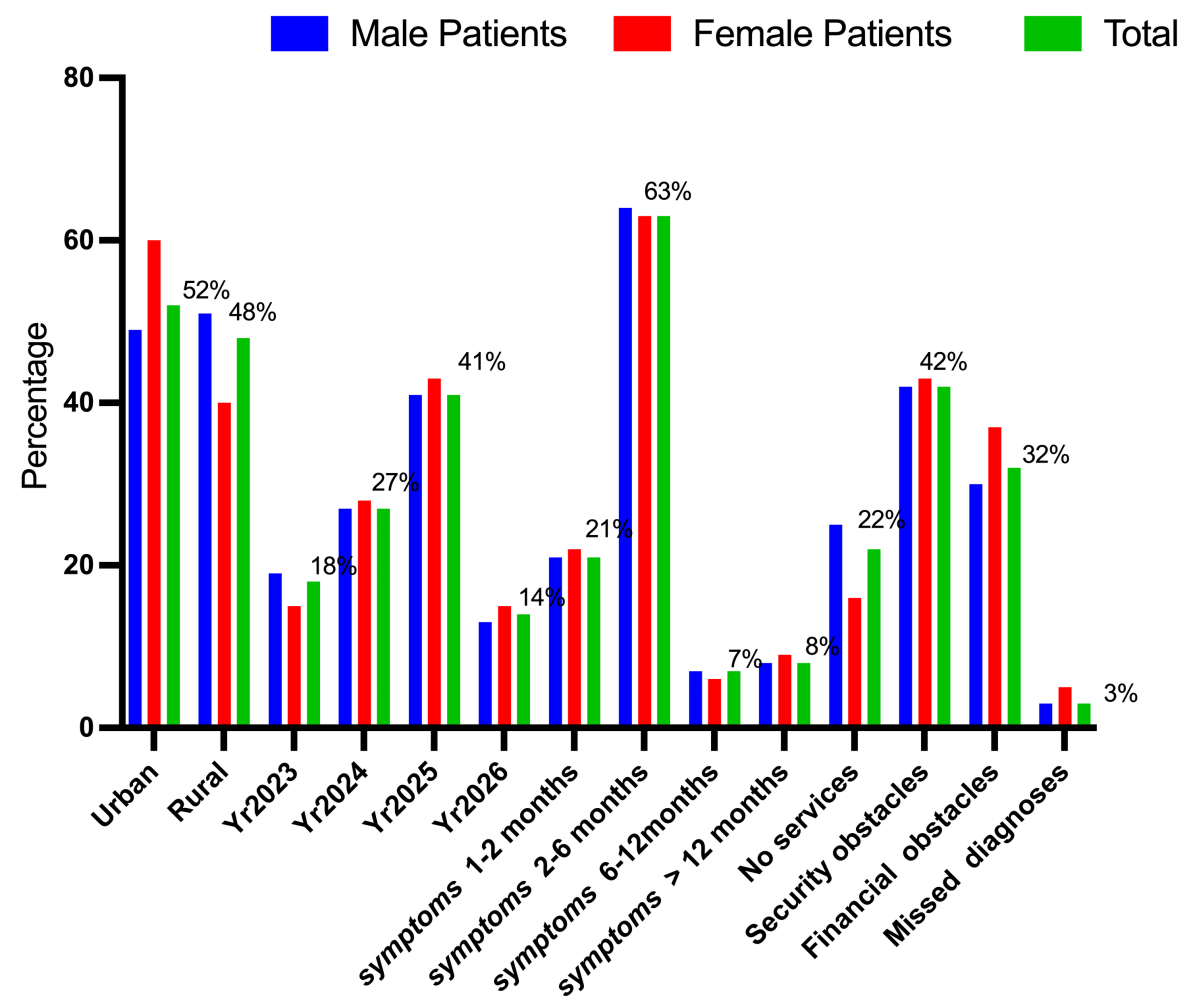
Description of patients by demographic features and characteristics of initial presentation

About 1389 (63%) of patients initiated anti-TB therapy after developing symptoms over two to six months. Factors responsible for the treatment delay include security obstacles (42%), financial obstacles (32%), a lack of health services in the patient's residence (22%), and missed diagnoses (3%), as shown in Table 1 and Figure 1.

**Table 1:** Distribution of patients by demographic features and characteristics of initial presentation

Variable	Male Patients N=1539	Female Patients N=654	Total N=2193
Residences			
Urban	760 (49.4%)	391 (60%)	1151 (52%)
Rural	779 (50.6%)	263 (40%)	1042 (48%)
Year of patients’ presentation			
2023	289 (18.8%)	97 (14.8%)	386 (18%)
2024	412 (26.8%)	180 (27.5%)	592 (27%)
2025	634 (41.2%)	279 (42.7%)	913 (41%)
2026	204 (13.3%)	98 (15%)	302 (14%)
Duration of symptoms			
1-2 months	324 (21%)	145 (22.2%)	469 (21%)
2-6 months	979 (63.6%)	410 (62.7%)	1389 (63%)
6-12months	109 (7.1%)	42 (6.4%)	151 (7%)
> 12 months	127 (8.3%)	57 (8.7%)	184 (8%)
Causes of delay in treatment			
No services	382 (24.8%)	103 (15.7%)	485 (22%)
Security obstacles	646 (42%)	282 (43%)	928 (42%)
Financial obstacles	466 (30.3%)	239 (36.5%)	705 (32%)
Missed diagnoses	45 (3%)	30 (4.6%)	75 (3%)

**Figure 2:**
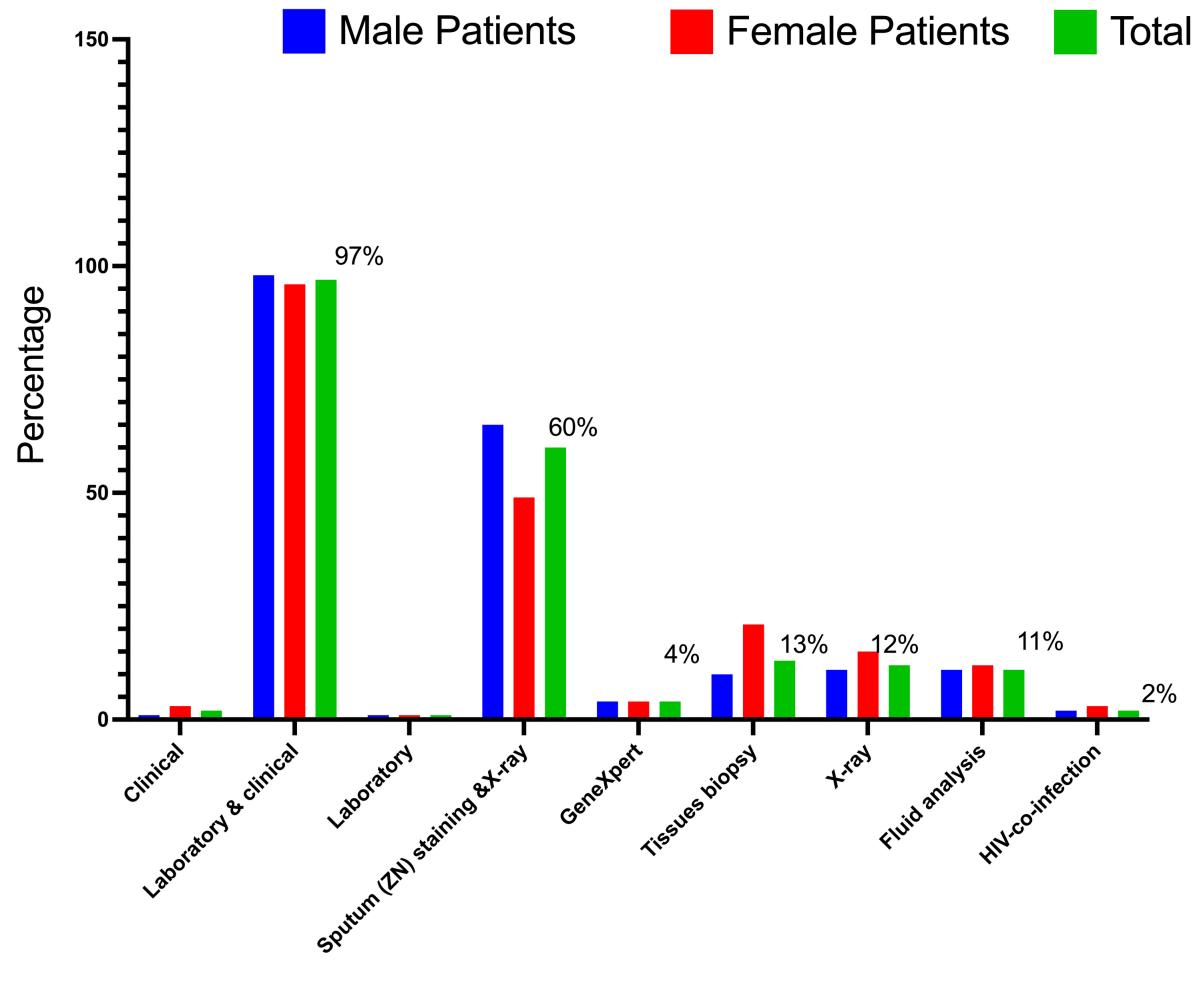
Description of patients by initial diagnosis, confirmatory diagnostic method, and coinfection

Most patients were initially diagnosed using combined clinical and laboratory approaches, followed by clinical and laboratory, representing 2135/2193 (97%), 43/2193 (2%), and 15/2193 (0.6%), respectively. The most confirmatory diagnostic tests used were combined sputum (ZN) staining & X-ray, followed by tissue biopsy, X-ray, fluid analysis, and GeneXpert, constituting 1314/2193 (60%), 286/2193 (13%), 268/2193 (12%), 239/2193 (11%), and 86/2193 (4%), in that order, as shown in Table 2 and Figure 2.

**Table 2:** Distribution of patients by initial diagnosis, confirmatory diagnostic method, and coinfection

Variable	Male Patients N=1539	Female Patients N=654	Total N=2193
Initial diagnosis			
Clinical	21 (1.4%)	22 (3.4%)	43 (2%)
Laboratory and clinical	1507 (98%)	628 (96%)	2135 (97%)
Laboratory	11 (0.6%)	4 (0.6%)	15 (0.6%)
Confirmatory diagnostic method			
Sputum (ZN) staining &X-ray	993 (64.5%)	321 (49%)	1314 (60%)
GeneXpert	61 (4%)	25 (3.8%)	86 (4%)
Tissues biopsy	152 (10%)	134 (20.5%)	286 (13%)
X-ray	170 (11%)	98 (15%)	268 (12%)
Fluid analysis	163 (10.6%)	76 (11.6%)	239 (11%)
Co. Infectious			
HIV-positive	26 (1.7%)	22 (3.4%)	48 (2%)

HIV coinfection was identified in 48 (2%) patients, including 26/1539 (1.7%) male patients and 22/654 (3.4%) female patients. The impact of co-infection with HIV in female patients is represented by an OR of 2.0257 (95% CI 1.1395 to 3.6010), p = 0.016, z-statistic = 2.405, as shown in Table 2 and Figure 2.

Regarding the patients’ presentation, most patients presented as a "new episode," followed by re-registered cases and MDR-TB cases, constituting 1986/2193 (90.6%), 194/2193 (8.8%), and 13/2193 (0.6%), respectively. Of the 13 MDR-TB cases, 6/2193(0.3%) were new episode, 5/2193(0.2%) were recurrent cases, and 2/2193(0.1%) were reregistered cases. Of the 194 reregistered cases, 109/2193 (5%) were recurrent cases, and 85/2193 (4%) were defaulter cases. The risk of re-registration among male patients, OR (95%CI) = 2.2066 (1.4995 to 3.2473), p = 0.0001, z statistics =4.015. Male patients have a rising rate of recurrent TB (OR (95%CI) = 2.0758 (1.2546 to 3.4347), p = 0.0045, z statistics = 2.843). Regarding health workers’ (healthcare staff) adherence to the treatment protocol (adherence to prescribing/guiding treatment protocols), about 2141/2193 (97.6%) adhered, and the remaining 52/2193 (2.4%) did not use the protocol. Of the 52 patients who were also non-adherent to the treatment protocol, most cited security concerns as the primary reason. Other factors included high transportation expenses, medication side effects, and the unavailability of medication in TMU, with respective occurrences of 24/2193 (1.1%), 12/2193 (0.5%), 9/2193 (0.4%), and 6/2193 (0.3%), as shown in Table 3.

**Table 3:** Distribution of the patients by sex and management characteristics MDR-TB: Multidrug-resistant tuberculosis

Variable	Male Patients N=1539	Female Patients N=654	Total N=2193
Patients’ presentation			
New episode	1372 (89%)	614 (94%)	1986 (90.6%)
Re-registered	161 (10.5%)	33 (5%)	194 (8.8%)
MDR-TB	6 (0.5%)	7 (1%)	13 (0.6%)
MDR-TB			
New episode	5 (0.3%)	1 (0.2%)	6 (0.3%)
Re-registered	2 (0.1%)	0 (0%)	2 (0.1%)
Recurrent	3 (0.2%)	2 (0.3%)	5 (0.2%)
Re-registered (for treatment)			
Failure	0 (%0)	0 (0%)	0 (0%)
Defaulter	70 (4.5%)	15 (2.3%)	85 (4%)
Recurrent cases	91 (5.9%)	18 (2.8%)	109 (5%)
Health workers adherent to the treatment protocol			
Adherent to the treatment protocol	1500 (97.5%)	641(98.6%)	2141 (97.6%)
Not adherent to the treatment protocol	39 (2.5%)	13 (2%)	52 (2.4%)
Poor patient adherence to the protocol			
No medication in TMU	5 (0.3%)	1 (0.2%)	6 (0.3%)
High transportation costs	9 (0.6%)	3(0.5%)	12 (0.5%)
Security reasons	17 (1.1%)	7 (1%)	24 (1.1%)
Incorrect beliefs about medication	0 (0%)	1 (0.2%)	1 (0.05%)
Medication side effects	6 (0.5%)	3 (0.5%)	9 (0.4%)

## Discussion

The distribution of TB cases in this study shows that most patients were detected and enrolled as new cases, with 90% falling into this category. This is a common pattern in many TB programs, indicating that routine case-finding and diagnostic procedures are capturing a high number of patients at their first presentation, rather than a largely chronically infected or previously treated population [9].

A lesser fraction of patients was designated as cases requiring re-treatment, amounting to 9%. In this cohort, instances of relapse represented 5%, while cases of default accounted for 4%. The occurrence of relapse cases indicates that a portion of patients who have previously undergone treatment subsequently experienced a resurgence of the disease. This situation may highlight concerns regarding the efficacy of the anti-tuberculous medications, potential reinfection, or insufficient monitoring following the conclusion of therapy. In a similar vein, the defaulter component underscores ongoing difficulties in maintaining treatment adherence and retention, as patients often disrupt their therapy and later present with TB once more. Collectively, these patterns of re-treatment underscore the necessity of enhancing post-treatment follow-up, bolstering adherence support, and implementing strategies to minimize missed appointments and premature discontinuation. The burden of conflict increases in low- and middle-income countries, where poverty, malnutrition, overcrowded living conditions, security barriers, and limited access to quality healthcare exist, which may signify the failure of treatment [10].

In terms of programmatic support, a significant majority of patients (98%) received follow-up from health workers regarding their treatment program, whereas a mere 52 out of 2193 (2%) did not. The extensive follow-up coverage observed is an encouraging outcome, indicating a robust connection between patients and health-worker oversight within the TB control framework. Consistent follow-up is essential for evaluating clinical response, maintaining adherence, managing side effects, and swiftly addressing any missed doses. A limited segment lacking follow-up could signify a demographic at increased risk for negative consequences, including treatment interruption, default, or postponed identification of complications. This situation necessitates focused interventions, such as bolstered community support, refined tracking systems, or the resolution of access barriers.

However, armed conflict can worsen TB transmission and disease severity, including population displacement, overcrowding in temporary settlements, health service disruption, reduced access to diagnostics and treatment, and nutrition and living conditions. Wartime delays in seeking care and starting treatment may perpetuate transmission in both displaced and host groups and increase clinically recognized cases.

The study also found that urban areas had the most TB cases. Cities' higher population density and intimate interaction may enhance TB respiratory dissemination. Urban locations may have better healthcare access than rural areas, increasing detection and reporting. Wartime displacement and migration may concentrate vulnerable groups in cities, increasing transmission and risk. The findings reveal a notable prevalence of HIV coinfection among patients diagnosed with TB [11]. Female HIV coinfection rates are far greater than male rates, which must be considered. The relative risk of HIV coinfection in women is 1.9912, with a 95% CI of 1.1370 to 3.4872 and a p-value of 0.016. This shows that HIV coinfection is roughly twice as likely in women as in men.

Different approaches are used to diagnose TB depending on the patient’s presentation. The present study shows that while traditional methods are still prominent, comprehensive diagnostic methodologies, especially for unusual TB presentations, are needed. Integrating sophisticated diagnostics and training for healthcare providers will improve TB detection, patient treatment, and TB control [12].

The low re-registration rate shows that treatment is effective, but adherence is difficult. Relapse rates and defaulter cases suggest socioeconomic factors, stigma, and lack of healthcare resources that may hinder treatment completion or effectiveness. The low rate of MDR TB is reassuring, although medication resistance trends must be monitored. Despite being rare in the patients of the current study, MDR TB requires specialized treatment, and its increase could threaten TB reduction efforts.

MDR-TB continues to pose a significant public health challenge. Certain regions, notably Eastern Europe, Central Asia, and regions in South and Southeast Asia, exhibit disproportionately elevated rates of resistance. MDR-TB is a variant of TB caused by bacteria resistant to rifampicin and isoniazid, the two primary first-line medications for TB treatment [13]. MDR-TB is treatable and curable with alternative medications, which are generally more costly and associated with increased side effects. Individuals exposed to MDR-TB may be administered preventive treatment with levofloxacin.

Detection of MDR-TB, as per WHO guidelines, necessitates bacteriological confirmation of TB and assessment for drug resistance through rapid molecular tests or culture techniques [14].

The fact that most patients have doctors follow their treatment plans is good. Healthcare practitioners must follow up on TB treatment regimens and evaluate side effects and consequences. Engaged health personnel can also help patients remember to finish their therapy to avoid relapses and transmission. Reaching these folks, who may face extra barriers to healthcare engagement, is crucial. The data provides valuable TB patient demographics and healthcare interaction information. Understanding trends in new cases, retreatment, and health worker follow-up helps assess TB care and supports public health policies to improve treatment adherence and control TB in the community. Successful TB treatment requires a comprehensive approach that prioritizes medical and socio-behavioral therapy [15-17].

Regarding patients’ adherence to the treatment protocol. Socioeconomic considerations strongly influence TB treatment and patient adherence. The research shows various distinct impediments to adherence, stressing the need for complete solutions.

Security issues may hinder patients from receiving treatment in some areas. Patients may fear assault or harassment while traveling to medical appointments or may not seek care. Patient transportation costs can be a major barrier, especially in low-income areas with inadequate or expensive public transit. Inability to pay may cause patients to miss visits or medicines.

TB drug side effects sometimes dissuade patients from taking their prescriptions. Long-term therapy side effects might cause discomfort or dread, forcing patients to discontinue taking their drugs. Patient adherence can be improved by educating them about side effects and providing supportive care. Lack of drugs at TB management facilities interrupts treatment continuity and can cause defaults. Maintaining medication supplies in TBMUs is crucial for patient adherence [18].

Strengths of this study

A sample of this size can reliably estimate proportions like delays, reasons for delay, and residence/sex distribution. Sex (70% male/30% female) and residence (52% urban/48% rural) assist in contextualizing delays and increasing interpretability.

Quantifying the proportion starting medicine within 2-6 months and identifying delay factors (security, financial, lack of resources, and missed diagnoses) improves public health measures. Reporting MDR breakdown (new vs MDR vs reregistered) and reregistered case composition (recurring vs defaulters) can improve the study's relevance to relapse and treatment interruption.

Limitations of the study

TB program datasets are often incomplete in this series due to war circumstances. The proportions may be biased if dates or categories (such as new, re-registered, or MDR) are missing or inconsistent. Patient reports, staff notes, and administrative issues frequently contribute to security, financial, and resource challenges. Additionally, results may not accurately represent community pathways or regional variations (for example, differing security conditions or referral systems) because the data is hospital-based and likely limited to specific facilities over a certain timeframe.

## Conclusions

The findings of the present study indicated increased frequencies of TB, particularly in the years 2025 and 2024, mainly among men. Coinfection with HIV was higher among women. Many patients started anti-TB medication late due to security challenges, financial constraints, and inadequate health-care services at the patients’ residences. New episodes dominated, followed by re-registered and MDR-TB cases. Recurrent cases and defaulters were high for re-registered patients, highlighting the role of treatment continuity and barrier difficulties. Overall, delayed treatment initiation driven largely by security and financial barriers remains a major contributor to TB burden, while MDR-TB and the high share of defaulters among re-registered cases call for improved early diagnosis, patient-centered support (including cost and adherence support), and follow-up systems to reduce poor outcomes and prevent drug resistance.
